# The effects of using sheep tail fat and cooking time on carboxymethyl‐lysine formation and some quality characteristics of heat‐treated sucuk

**DOI:** 10.1002/fsn3.4067

**Published:** 2024-03-07

**Authors:** Pınar Anlar, Güzin Kaban

**Affiliations:** ^1^ Department of Food Technology, Vocational College of Technical Sciences Atatürk University Erzurum Turkey; ^2^ Department of Food Engineering, Faculty of Agriculture Atatürk University Erzurum Turkey

**Keywords:** AGE, carboxymethyl‐lysine, fermented sausage, heat‐treated sucuk, sheep tail fat

## Abstract

The study's aim was to determine the effect of using sheep tail fat (STF) on carboxymethyl‐lysine (CML) content and other properties of heat‐treated sucuk (HTS), a type of semi‐dry fermented sausage. Three mixtures were prepared: 100% beef fat (BF), 50% BF + 50% STF, and 100% STF. After production (fermentation, heat treatment, and drying), the samples were cooked at 180°C for 0, 1, 3, and 5 min to determine the effect of cooking time on CML, thiobarbituric acid reactive substance (TBARS), total sulfhydryl, and carbonyl contents. The lowest pH value (5.50) was observed in the presence of STF. The most oleic acid (46.02%) was observed in the 100% STF group. The score of taste and general acceptability decreased with increasing STF. Using STF had no significant effect on TBARS, total sulfhydryl, carbonyl, or CML content. These parameters were affected by cooking time. The mean CML content increased from 55.77 to 72.90 μg/g after 5 min of cooking. CML correlated more strongly with TBARS than sulfhydryl or carbonyl.

## Graphical abstract

Sheep tail fat affected pH, aw, NPN‐S, color, and fatty acid composition. Sheep tail fat did not affect CML, TBARS, sulfhydryl, carbonyl contents. Cooking increased significantly CML, TBARS, sulfhydryl, carbonyl contents. Sheep tail fat caused undesirable sensory properties in heat‐treated sucuk.

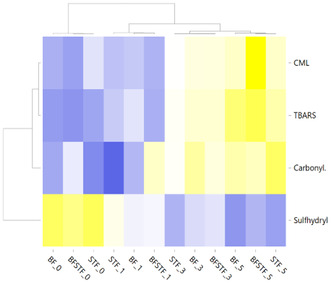

## 1 INTRODUCTION

The Maillard reaction, which causes the formation of desirable and undesirable compounds in foods (Nursten, 2005; Vlassara & Uribarri, 2004), starts between the carbonyl group of reducing sugars and the amino group or free amino groups of amino acids (Chen et al., 2017; Rezvankhah et al., 2024). As the reaction progresses, Amadori products are formed. Then, along with condensation, dehydration, and decomposition reactions, advanced glycation end products (AGEs), risky for health, are formed (Solís‐Calero et al., 2015; Wei et al., 2018).

Diet is the most important source of exogenous AGEs (Chen & Smith, 2015; He et al., 2014; Wei et al., 2018), but they can also be produced endogenously in the body (Kellow et al., 2018). Among AGEs, carboxymethyl‐lysine (CML) is an accepted indicator of glycoxidation and lipoxidation (Arena et al., 2013). The fact that most AGEs are not as stable as CML in acidic conditions causes (Chen & Smith, 2015) CML to be frequently used as an AGE indicator in foods (Zhu, Fang, et al., 2020; Zhu, Huang, et al., 2020). There are two important CML formation pathways in foodstuffs: the oxidation of fructosyl lysine and the reaction of lysine with the ε‐amino group via the advanced lipoxidation end products (ALE) pathway (Delgado‐Andrade, 2015).

In the formation of CML in food, oxidation of lipids plays an important role because it reduces sugar auto‐oxidation, polyol degradation, and reactive carbonyl species (methylglyoxal, polyol dicarbonyls, malondialdehyde) (Zhu, Fang, et al., 2020; Zhu, Huang, et al., 2020). The development of surface color and flavoring cooked meat and meat products is mainly due to the Maillard reaction and the interactions between lipid oxidation products (malondialdehyde, 2,4‐decadienal, hexanal, etc.) and both reaction pathways (Roldan et al., 2015). Oxidation of protein in meat processing may also play a role in the formation of AGEs, but this has not been sufficiently clarified (Zhu, Fang, et al., 2020; Zhu, Huang, et al., 2020).

There are studies on CML in different sausage types such as dried sausage, cooked sausage, and fermented sausage (Li et al., 2023; Lu et al., 2023; Wang et al., 2023; Yu et al., 2018, 2022). One study investigated the effects of processing conditions, fat content, and additives on CML formation in cooked sausage (Lu et al., 2023). However, there have been no studies on CML in heat‐treated sucuk (HTS), a beef sausage widely consumed in Türkiye. HTS is a semi‐dry fermented sausage. Its preparation involves fermentation, heat treatment, and drying (Kaban, Bayraktar, et al., 2022). It is usually fried or grilled before eating (Akansel et al., 2023; Sallan et al., 2020), so CML may be an important contaminant in HTS. Beef fat is generally used (Soyer et al., 2005; Yalınkılıç et al., 2016), but sheep tail fat (STF) can also be used, either on its own (Kaban, 2013; Sarıçoban et al., 2005), or combined with beef fat (Kaban, Bayraktar, et al., 2022). STF is important because it is cheaper than other animal fats (Aktaş & Gençcelep, 2006) and contains high amounts of unsaturated fatty acids (Şişik Oğraş et al., 2018; Ünsal & Aktaş, 2003).

This study's main aim was to investigate the effects of STF (100% beef fat (BF), 50% BF + 50% STF, and 100% STF) and cooking time (at 180°C for 0, 1, 3, and 5 min) on CML, TBARS, total sulfhydryl, and carbonyl content of HTS. The secondary aim was to determine the effect of STF on other characteristics (microbiological, physico‐chemical, and sensory properties).

## 2 MATERIALS AND METHODS

### 2.1 Materials

Meat from the shoulders of three‐year‐old male cattle carcasses (after 24 h postmortem at 4°C ± 1°C) was purchased from the local slaughterhouse (Erzurum, Türkiye). Excess fat and connective tissues were removed. Sheep tail fat (STF) and beef fat (BF) were used as fat. Garlic, spices, and salt were purchased from local markets in Erzurum. Sodium nitrite was used as the curing agent, and the meat batters were placed in collagen casing (38 mm, Naturin, Weinheim, Germany). *Staphylococcus xylosus* GM92 (Kaban & Kaya, 2009) and *Lactobacillus sakei* S15 (Kaban, Sallan, et al., 2022) strains were used as starter cultures in production.

### 2.2 Heat‐treated sucuk (HTS) production

Three batches of HTS were independently prepared, and three groups were prepared in each batch: 100% BF (control), 50% BF + 50% STF, and 100% STF. A total of 9 batters were prepared, following Armutcu et al. (2020). Batters were prepared with a cutter (Mado, Schwarzwald, Germany) using lean beef (80%) and fat (20%). For each kg of meat and fat, 7 g of red pepper, 4 g of sucrose, 2.5 g of allspice, 9 g of cumin, 5 g of black pepper, 10 g of garlic, 20 g of NaCl, and 0.15 g of sodium nitrite were added. *S. xylosus* GM92 and *L. sakei* S15 strains were inoculated with approximately 10^6^ and 10^7^ CFU/g, respectively. After preparation, the batters were placed into collagen casings using a filling machine (Mado 591, Schwarzwald, Germany). The fermentation stage was continued until the sample pH fell to 5.3 in a climate chamber (Reich, Schechingen, Germany) at 24 ± 1°C and 90 ± 2% relative humidity (RH). HTS samples were then heat treated in a steam cooking unit (Mauting, Czech Republic) at 68°C core temperature. The samples were dried for 3 days in the climate unit (80 ± 2% RH, 16 ± 1°C).

### 2.3 Analyses

The prepared samples were analyzed for pH, water activity (a_w_), L*, a*, b*, non‐protein nitrogenous substances (NPN‐S), fatty acid composition, and sensory and microbiological characteristics. Samples from each group were sliced into 0.5 cm thick slices and cooked on a hot plate at 180°C for 0, 1, 3, and 5 min, and thiobarbituric acid reactive substances (TBARS), total sulfhydryl, carbonyl, and carboxymethyl‐lysine (CML) were determined.

#### 2.3.1 Microbiological analysis

Lactic acid bacteria were counted on De Man Rogosa Sharpe Agar plates (Merck, Darmstadt, Germany). Enterobacteriaceae was counted on Violet Red Bile Dextrose Agar (Merck) plates. Samples were incubated anaerobically at 30°C for 2 days. *Micrococcus/Staphylococcus* were enumerated on Mannitol Salt Phenol Red Agar (Merck), and the plates were incubated at 30°C for 2 days (Baumgart et al., 1993).

#### 2.3.2 Determination of pH and water activity (a_w_), non‐protein nitrogenous substances (NPN‐S) content, and instrumental color

Analyses were done with a pH meter with automatic thermal compensation (Gökalp et al., 2010) and an a_w_ meter (Novasina, TH‐500 aw Sprint). Non‐protein nitrogenous substances (NPN‐S) contents were detected following Anonymous (1989). Dichloromethane and 20% trichloroacetic acid solution were added to 4 g of sample. Homogenization was done using an Ultra‐Turrax (IKA, Germany) for 30 s; the homogenate stood at ambient temperature for 15 min. The homogenate was centrifuged (Beckman Coulter, USA) and then filtered with Whatman 595 filter paper. Twenty grams of the filtrate was transferred to a Kjeldahl flask. Based on the Kjeldahl method, the total nitrogen contents of the HTS samples were determined, and the results were given as g/100 g (%) of the sample. L*, a*, and b* values were detected with a colorimeter (CR 400, Minolta, Japan) with a *C D65 illuminator, 8 mm hole size, and 2° observation standard.

#### 2.3.3 Determination of thiobarbituric acid reactive substances (TBARS), total sulfhydryl, and carbonyl contents

TBARS values were determined according to Lemon (1975), and the results are given as μmol MDA/kg.

Sulfhydryl amounts were determined by considering the 5,5′‐dithiobis (2‐nitrobenzoic acid) reaction according to Vossen and De Smet (2015) and Lund et al. (2008). According to the method, after weighing a 2 g sample, 0.1 M Tris buffer (pH 8.0) containing 5% sodium dodecyl sulfate was added. Homogenization was carried out with an Ultra‐Turrax. Then, the samples were kept in a water bath (30 min at 80°C) and centrifuged (20 min at 10,000 g). After the centrifuged samples were filtered, their protein content was adjusted to 1.5 mg/mL. The absorbance values of the samples were determined at 412 nm against the reference, after being kept in the dark at ambient temperature for 30 min (Ellman, 1959). The extinction coefficient (Ec) of 14,000/M/cm was used to calculate the sulfhydryl content.

Carbonyl amounts in HTS were assessed following Oliver et al. (1987). One gram of the sample was weighed and homogenized with 0.15 M KCl (10 mL); 50 μL of the homogenized sample was taken and added to tubes containing 10 mM DNPH (2,4‐Dinitrophenylhydrazine). After adding 1 mL of TCA (20%) to the tubes, they were left at ambient temperature for 1 h and the samples were centrifuged (10 min at 278 *g*; Beckman Coulter, Allegra X‐30R) to remove the supernatant. Then, samples were washed with 1 mL of ethanol: ethyl acetate (1:1) solution until the color turned completely white and the supernatant was removed. 6 M guanidine + sodium phosphate buffer (2 mL) was added to the samples, dried with nitrogen, and kept at 37°C for 15 min. The absorbances of the samples were determined against 6 M guanidine + sodium phosphate buffer at 370 nm. An extinction coefficient of 21,000/M/cm was used to calculate the carbonyl content of the samples.

#### 2.3.4 Determination of CML content

After 10 μL of hydrolyzate was mixed with 40 μL of orthophthaldehyde and left for 5 min, the content was determined using HPLC (Agilent Technologies, USA). TSK gel ODS‐80 TM (25 cm × 4.6 mm, 5 μm, 80A, USA) column and fluorescence detector were used. Acetate buffer/acetonitrile (90:10, v/v) was used as the mobile phase, and a flow rate of 1.0 mL/min was set as an injection volume of 20 μL. The CML standard (Cayman Chemical, Michigan, USA) was used for identification (Bayrak Kul et al., 2021).

The recovery rates were determined five times at five different levels (1, 5, 10, 20, and 25 μg/mL) using the spike samples with the CML standard. The range of mean recoveries was 99.40%–104.02% with relative standard deviations of 1.21%–4.53%. The coefficient of the regression line was 0.9999, calculated with the formulas LOQ = 10 × Sy/s and LOD = 3.3 × Sy/s. LOQ was 1.62 μg/mL and LOD was 0.53.

#### 2.3.5 Determination of fatty acid composition

The fats of HTS samples were obtained following Folch et al. (1957), and then methylation was carried out according to Metcalfe and Schmitz (1961). Chloroform was removed with a rotary evaporator, and NaOH with methanol was added to the remaining fat and treated with nitrogen after transferring it to the sample tube. The samples were dried at 80°C and cooled. BF3‐methanol was added to the cooled sample, treated with nitrogen again, dried (at 80°C for 30 min), and cooled. Hexane (1 mL), distilled water (1 mL), and hexane (1 mL) were added and mixed. Samples were centrifuged, and the resulting supernatant was transferred to a tube containing anhydrous sodium sulfate using a Pasteur pipette and mixed. After phase separation, the clear upper phase was taken into a vial and treated with nitrogen. The compositions of HTS samples were determined using GC/FID (Agilent Technologies 6890 N, USA). CPSIL88 column (Agilent, 100 m × 250 μm × 0.20 μm) was used, and the carrier gas was helium. The identification was performed using a fatty acid methyl ester mixture (Supelco, FAME‐mix, PA, USA).

### 2.4 Sensory analysis

Sensory analysis was carried out with 20 semi‐trained panelists in the field of Food Engineering (Atatürk University, Erzurum, Türkiye), who were informed about the sensory analysis process and scorecard. A nine‐point hedonic‐type scale was used. The HTS samples were cut into 1 cm‐thick slices and prepared for analysis at room temperature. Three replicates were tested per treatment following a randomized complete block design with three samples per plate. Three replicates of each sample were evaluated separately by the same panelists.

### 2.5 Statistical analysis

A total of three HTS groups were made in each batch, and experiments were performed with a randomized complete block design. Two random samples were taken from each batch, and analyses were performed in duplicate. Data were analyzed with an analysis of variance (ANOVA) using a general linear model, treating STF as a main effect and the replicates as random effects. In addition, cooking time (0, 1, 3, and 5 min) was also considered in CML, TBARS, carbonyl, and sulfhydryl contents as a main effect (3 × 4 factorial design). Significant sources of variation were compared by means of Duncan's multiple comparison test (SPSS 24, Chicago, IL, USA). A heatmap was made to visualize the relationship between TBARS, carbonyl, sulfhydryl, and CML contents, and the HTS groups (Babicki et al., 2016).

## 3 RESULTS AND DISCUSSION

### 3.1 Microbiological properties

Producing HTS involves heat treatment, and the core temperature was varied between 60–68°C in this stage (Yılmaz Oral & Kaban, 2023a). Lactic acid bacteria (LAB) growth was up to 10^8^ CFU/g during the fermentation stage. Heat applied after this stage causes significant reductions in the number of LAB and *Micrococcus/Staphylococcus*, depending on the internal temperature (Armutcu et al., 2020; Çakır et al., 2013; Yılmaz Oral & Kaban, 2021). In the present study, the number of LAB, *Micrococcus/Staphylococcus*, and Enterobacteriaceae were below <2 log CFU/g, due to the application of a core temperature of 68°C in the heat treatment stage (data not shown).

### 3.2 pH, a_w_, instrumental color values, and non‐protein nitrogenous substances (NPN‐S) content

Table 1 shows the effect of using STF on pH, a_w_, and L*, a* and b* values, and non‐protein nitrogenous substances (NPN‐S) content. The lowest mean pH value was 5.50 in the group with 100% STF. According to the Turkish Food Codex Communiqué on Meat, Prepared Meat Mixtures, and Meat Products (Anonymous, 2019), the pH value in HTS should not exceed 5.6. In our study, all pH values were below 5.6. The lowest mean a_w_ value (0.917) was in the groups with 100% BF, and this group differed statistically from the 100% STF group. These results are similar to others on HTS (Armutcu et al., 2020; Çakır et al., 2013; Yılmaz Oral & Kaban, 2023b).

**TABLE 1:** The effect of using STF on pH, a_w_, NPN‐S, L*, a*, and b* values of HTS.

Fat type	pH	*a*_w_	NPN‐S (g/100 g)	L*	a*	b*
BF	5.56 ^a^	0.917^b^	1.47^c^	45.87^a^	16.32^ab^	15.74^b^
BF + STF	5.54^a^	0.923^ab^	1.55^b^	43.15^b^	17.33^a^	16.54^a^
STF	5.50^b^	0.928^a^	1.69^a^	44.19^ab^	15.88^b^	17.17^a^
Significance	**	**	**	*	*	**
SE	0.01	0.00	0.01	0.36	0.23	0.16

Abbreviations: a*, redness; b*, yellowness; BF, beef fat; HTS, heat treated sucuk; L*, brightness; NPN‐S, non‐protein nitrogenous substances; SE, standard error of the mean; STF, sheep tail fat.

^a‐c^Means marked with different letters in the same column are statistically different from each other (*p* < .05).

**p < .01, *p < .05.

The lowest mean NPN‐S was in the 100% BF group; the highest was in the 100% STF group (Table 1). Proteolysis is one of the important biochemical changes that occur during sausage fermentation (Eim et al., 2008), and as time increases, the amount of small molecular weight compounds such as free amino acids and peptides also increases (Kaban & Kaya, 2009). As a result of proteolysis in fermented meat products, the amount of NPN and therefore NPN‐S increases. In the current study, NPN‐S content varied between 1.47 and 1.69. This result is due to the fact that this product, which is a kind of semi‐dry fermented sausage, has a short production period. In addition, the process of HTS includes heat treatment. On the other hand, high NPN‐S values were detected in sucuk, a dry‐fermented sausage. In the present study, using STF had a significant effect on NPN‐S value; the lowest NPN‐S value was in the STF group. It is thought that this is related to pH (Table 1) since it has been reported that proteolysis occurs more slowly in low‐acid sausages (Kaban & Kaya, 2009).

Using different amounts of STF led to a decrease in L* and a* and an increase in b* (Table 1). Another study conducted on sucuk also reported that the samples with STF showed a lower mean L* than the group with beef fat, with no differences between a* and b* (Sallan, 2023). In the color formation of fermented meat products, myoglobin combines with nitric oxide, which is formed due to the breakdown of nitrite, to form nitrosomyoglobin. Later, with the heat treatment applied in HTS production, nitrosohemochrome is formed, resulting in the stable pinkish‐red cured color of HTS.

### 3.3 TBARS, total sulfhydryl, and carbonyl content

The use of STF did not have a significant effect on TBARS, but cooking time did have a very significant (*p* < .01) effect. TBARS increased as cooking time increased in all groups (Table 2). However, it has been reported that the degree of oxidation may vary depending on the cooking method and time (Alfaia et al., 2010; Juarez et al., 2010; Roldan et al., 2014).

**TABLE 2:** The effects of using STF and cooking time on TBARS, total sulfhydryl, carbonyl, and CML contents of HTS.

Fat type (FT)	TBARS (μmol MDA/kg)	Total sulfhydryl content (μmol/mg protein)	Carbonyl content (nmol/mg protein)	CML (μg/g)
BF	18.12	22.26	3.85	60.90
BF + STF	17.90	22.39	4.14	64.49
STF	17.31	22.40	3.52	61.26
Significance	ns	ns	ns	ns
Cooking time (CT; min)				
0	14.57^d^	29.38^a^	3.06^b^	55.77^c^
1	16.04^c^	22.33^b^	3.23^b^	56.08^c^
3	18.76^b^	20.00^bc^	4.26^a^	64.11^b^
5	21.74^a^	17.70^c^	4.79^a^	72.90^a^
Significance	**	**	**	**
FT × CT	ns	ns	*	ns
SE	0.23	0.46	0.13	1.22

Abbreviations: BF, beef fat; HTS, heat treated sucuk; SE, standard error of the mean; STF, sheep tail fat; TBARS, thiobarbituric acid reactive substance.

^a‐d^Means marked with different letters in the same column are statistically different from each other (*p* < .05), **p < .01, *p < .05.

In this study, although the use of STF had no significant effect on the total sulfhydryl content, cooking time did affect the total sulfhydryl content of the HTS samples (*p* < .01) (Table 2). Heat treatment is one of the most important factors affecting protein oxidation since high‐temperature applications cause the denaturation of proteins (Dominguez et al., 2022). The highest total sulfhydryl content (29.38 μmol/mg protein) was in HTS without additional cooking (0 min). The degree of protein oxidation was the greatest in the 5 min heat treatment, however, this value did not differ from the mean after 3 min of cooking (Table 2). This result suggests that protein oxidation has a different trend than lipid oxidation. As a result of protein oxidation, modifications in the amino acid side chain and cross‐linking of different proteins may occur, as well as breaks in peptide bonds (Estevez, 2011). Free radicals cause the oxidation of thiol (‐SH) groups of sulfur‐containing amino acids in proteins and sulfhydryl groups (‐SH) are converted to other oxidized products together with disulfides (S‐S), resulting in a decrease in their amount (Dean et al., 1997).

**FIGURE 1:**
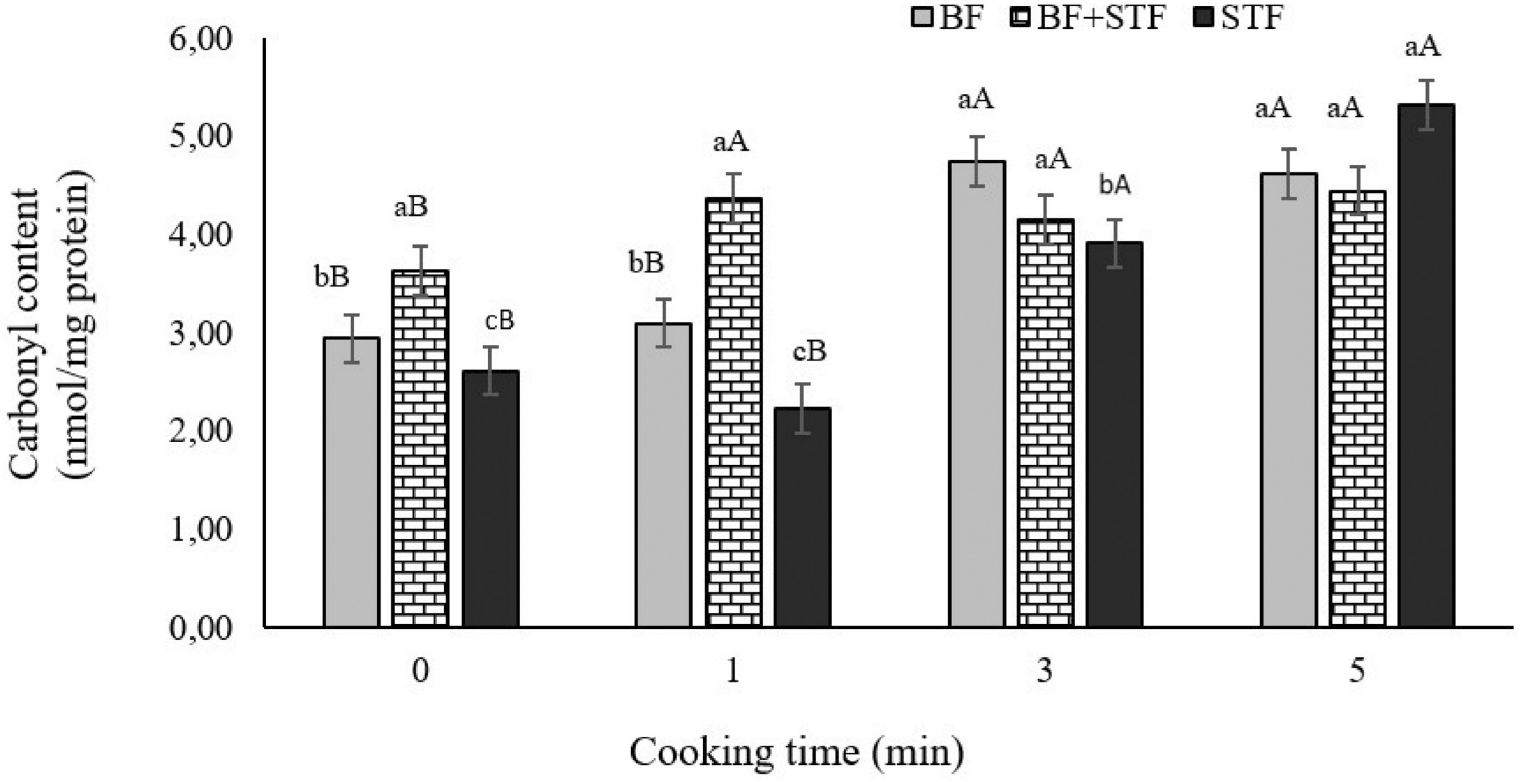
The interaction of using STF and cooking time on carbonyl content of heat‐treated sucuk. (a‐c): Different small letters indicate significant differences between the groups. (A‐B): Different capital indicates significant differences between cooking times for groups.

Carbonyl content is another important indicator of oxidative damage of proteins. Similar to total sulfhydryl results, the use of STF had no significant effect on carbonyl content, but cooking had a very significant effect (*p* < .01; Table 2). Carbonyl content increased with cooking time. The highest content (4.79 nmol/mg protein) was for 5 min of cooking time, with no statistical difference between 3 and 5 min of cooking (Table 2). The interaction of STF and cooking time had a significant effect (*p* < .05) on carbonyl values (Figure 1). In HTS groups cooked for 0, 1, and 3 min, STF had lower average values than the other groups (BF and BF + STF). On the other hand, in the HTS groups cooked for 5 min, there were no significant differences between the samples. In samples containing STF and BF, cooking for 3 and 5 min showed higher values than cooking for 0 and 1 min. According to these results, the carbonyl content in the samples containing STF + BF increased after 1 min of cooking and there was no statistical change thereafter. In the presence of STF and BF, the carbonyl content increased after 3 min of cooking time, and no change was observed thereafter (Figure 1). In contrast, it was reported that the use of olive oil in HTS production caused an increase in carbonyl content (Zungur et al., 2015).

### 3.4 Carboxymethyl‐lysine

The use of STF had no significant effect on the CML content of HTS (Table 2). On the other hand, it was reported that STF increased CML content in meatballs cooked at 180°C (dry heating), and this result was associated with the TBARS value. Furthermore, it was emphasized that STF is more effective in CML formation as cooking time increases (Öztürk et al., 2023). In the present study, cooking time had a very significant effect on CML content (*p* < .01). However, there was no interaction between the usage of STF and CML content (Table 2). The highest CML content was determined for 5 min cooking time (*p* < .05), and the cooking for 1 min had no significant effect on the CML content (*p* > .05; Table 2). Other studies have also shown that CML reflects heat treatment intensity (Chen & Smith, 2015; Öztürk et al., 2023; Sun et al., 2015; Trevisan et al., 2016; Zhu, Fang, et al., 2020; Zhu, Huang, et al., 2020). The formation of CML is influenced by factors such as meat type, fat type, and ingredients (Chen & Smith, 2015; Öztürk et al., 2023; Zhu, Fang, et al.,&nbsp;^2020^; Zhu, Huang, et al., 2020). In the current study, using STF had no influence on TBARS, total sulfhydryl, carbonyl contents, or CML content (Table 2).

### 3.5 Heatmap analysis

**FIGURE 2:**
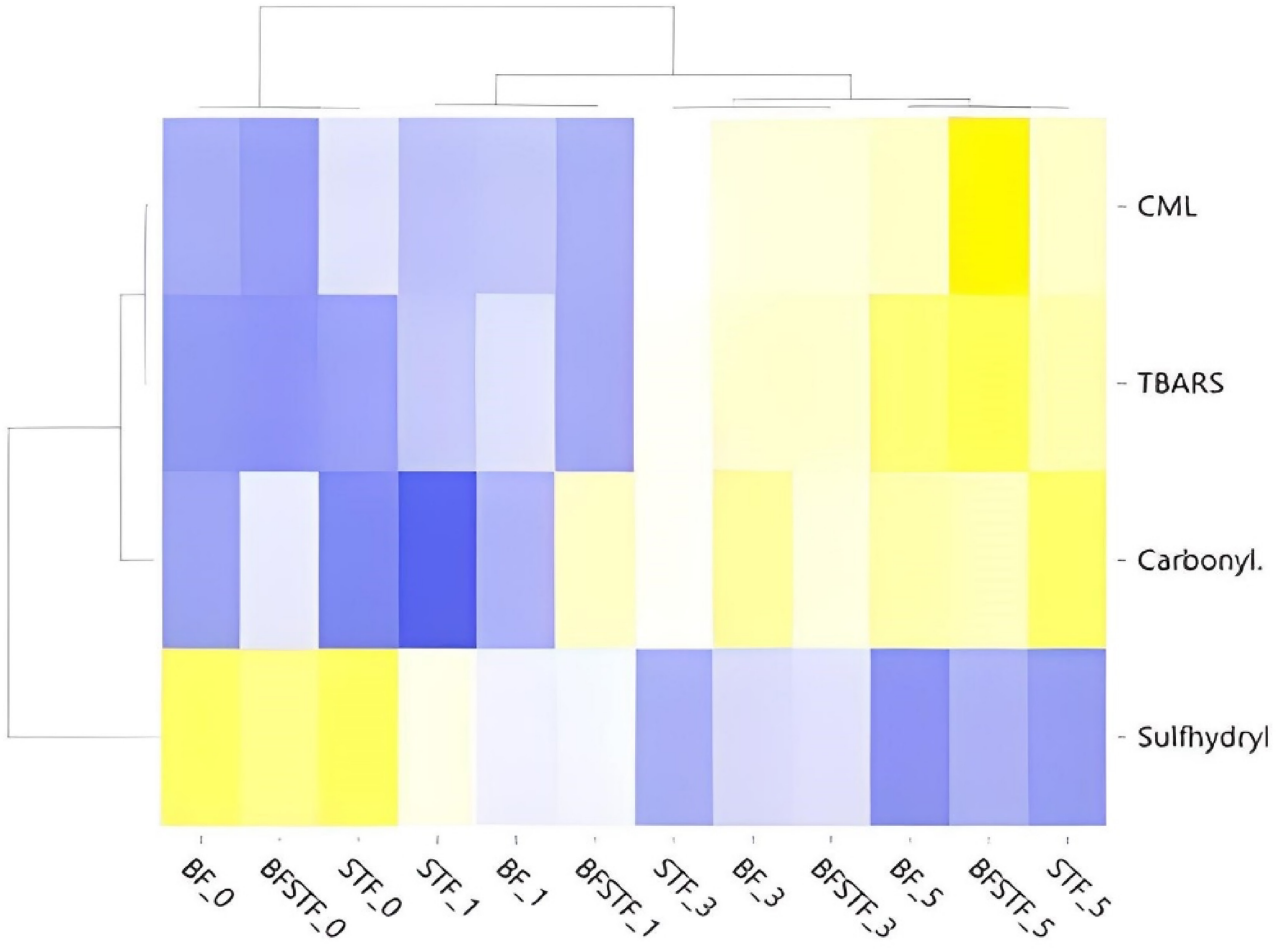
Cluster analysis of heatmap showing the relationship between carboxymethyl‐lysine, TBARS, sulfhydryl, carbonyl contents, and the samples containing different types of fat and cooked at different times (The intensity of the yellow and blue color represents from higher to lower correlation levels).

Cluster analysis was used to investigate the differences between HTS groups (BF, BF + STF, and STF), and cooking times with the variables CML, TBARS, total sulfhydryl content, and carbonyl content (Figure 2). Low, middle, and high expression levels are shown by colors in the heatmap. The heatmap uses the Pearson correlation for measuring distance and average connectivity. Figure 2 shows two main clusters of samples comprising different HTS groups and cooking times. The first cluster included only uncooked samples in all groups. The second cluster had two sub‐groups: in the first, samples cooked for 1 min, and in the second, samples cooked for 3 and 5 min. CML, TBARS, total sulfhydryl content, and carbonyl content were more affected by cooking time; the use of STF was not important for these parameters. For these parameters, there were two main clusters. The first included only sulfhydryl content. The second cluster has two subgroups, one with carbonyl content and the second with CML and TBARS. The Pearson correlation heatmap shows that while STF did not correlate with CML, cooking time, especially 3 and 5 min, did correlate strongly with these parameters. CML content correlated more strongly with TBARS, an indicator of lipid oxidation, than sulfhydryl and carbonyl content (Figure 2). Bayrak Kul et al. (2021) also reported a relationship between CML and TBARS in a traditionally cooked meat product.

### 3.6 Fatty acid profile

Table 3 shows the effect of STF on the fatty acid profile (%) of HTS. The HTS group produced with only STF has more myristoleic acid (C14:1) and oleic acid (C18:1n9c), which are unsaturated fatty acids (USFA) (Table 3). In another study of a cooked meat product, it was reported that using STF increases oleic acid (Şişik Oğraş et al., 2018). In the current study, there were some differences between groups for other fatty acids, but using STF had no effect on palmitoleic acid (C16:1) or linoleic acid (C18:2n6c; Table 3).

**TABLE 3:** The effect of using STF on fatty acid profile (%) of HTS.

Fatty acid	BF	BF + STF	STF	Significance	SE
C14:0	8.18^b^	11.25^a^	7.96^b^	*	0.46
C14:1	1.30^a^	1.25^a^	0.51^b^	**	0.03
C16:0	28.19^a^	24.43^ab^	22.83^b^	*	0.76
C16:1	5.42	5.01	3.75	ns	0.30
C18:0	14.82^b^	18.48^a^	14.94^b^	*	0.53
C18:1n9c	37.62^b^	36.03^b^	46.02^a^	*	1.19
C18:2n6c	4.47	3.55	4.00	ns	0.25

Abbreviations: BF, beef fat; HTS, heat treated sucuk; ns, not significant; SE, standard error of the mean; STF, sheep tail fat.

^a,b^Means marked with different letters in same column are statistically different from each other (*p* < .05), **p < .01, *p < .05.

### 3.7 Sensory analysis

Table 4 shows the effect of using STF on HTS sensory properties. STF had no significant effect on color or texture parameters. However, it did have an effect on odor (*p* < .05) as well as taste and general acceptability (*p* < .01). The STF group had the lowest scores for taste and general acceptability; the all‐beef (BF) group had the highest scores (Table 4). These results suggest that using STF in HTS production has a generally negative effect on sensory properties.

**TABLE 4:** The effect of using STF on sensory analysis of HTS.

Parameter	BF	BF + STF	STF	Significance	SE
Color	7.48	7.44	7.31	ns	0.10
Texture	7.36	7.07	6.67	ns	0.13
Odor	7.57^a^	6.88^ab^	6.65^b^	*	0.14
Taste	7.59^a^	6.69^b^	6.00^c^	**	0.10
General acceptability	7.61^a^	6.88^b^	6.19^c^	**	0.10

Abbreviations: BF, beef fat; HTS, heat treated sucuk; ns, not significant; SE, standard error of the mean; STF, sheep tail fat.

^a‐c^Means marked with different letters in the same column are statistically different from each other (*p* < .05), **p < .01, *p < .05.

## 4 CONCLUSION

The use of STF in HTS production did not cause a change in microbiological properties. At the same time, the changes in the physicochemical properties of the product such as pH, a_w_, and color were not significant enough to affect the characteristic features of the product. While using STF had no effect on CML, TBARS, sulfhydryl, or carbonyl content, which are indicators of protein oxidation, cooking time stood out as a very important factor. Hence it is thought that HTS, which is a ready‐to‐eat product, should not be subjected to heat treatment for more than 1 min. It should also be taken into account that STF leads to significant reductions in taste and general acceptability parameters.

## AUTHOR CONTRIBUTIONS

**Pınar Anlar:** Conceptualization (equal); formal analysis (lead); investigation (equal); methodology (equal); writing – original draft (lead). **Güzin Kaban:** Conceptualization (equal); investigation (equal); methodology (equal); project administration (lead); supervision (lead); validation (lead); writing – review and editing (lead).

## FUNDING INFORMATION

This research has received no specific funding from the public, commercial, or not‐for‐profit sector.

## CONFLICT OF INTEREST STATEMENT

The authors declare that they have no conflicts of interest of any kind.

## Data Availability

Data supporting the findings of this study are available on request from the corresponding author.
